# Supplementary material for: Design of a Novel and Potent Multi-Epitope Chimeric Vaccine against Human Papillomavirus (HPV): An Immunoinformatics Approach
Source: Biomedicines. 2023 May 22;11(5):1493. doi: 10.3390/biomedicines11051493 (PMC10216030; doi:10.3390/biomedicines11051493)
Supplement: Supplementary file 1 [file biomedicines-11-01493-s001.zip › biomedicines-2376147-supplementary.pdf]

Supplementary Materials

Article

Design of a Novel and Potent Multi-Epitope Chimeric Vaccine against Human Papillomavirus (HPV): An Immunoinformatics Approach

Muhammad Shahab [1,†], Dejia Guo [2,†], Guojun Zheng [1,*,†] and Yening Zou [2,*,†]

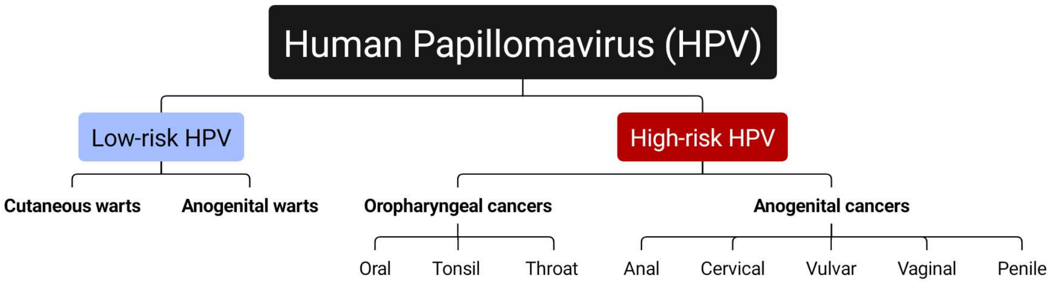

Figure S1. Classification of HPV viral types based upon severity.

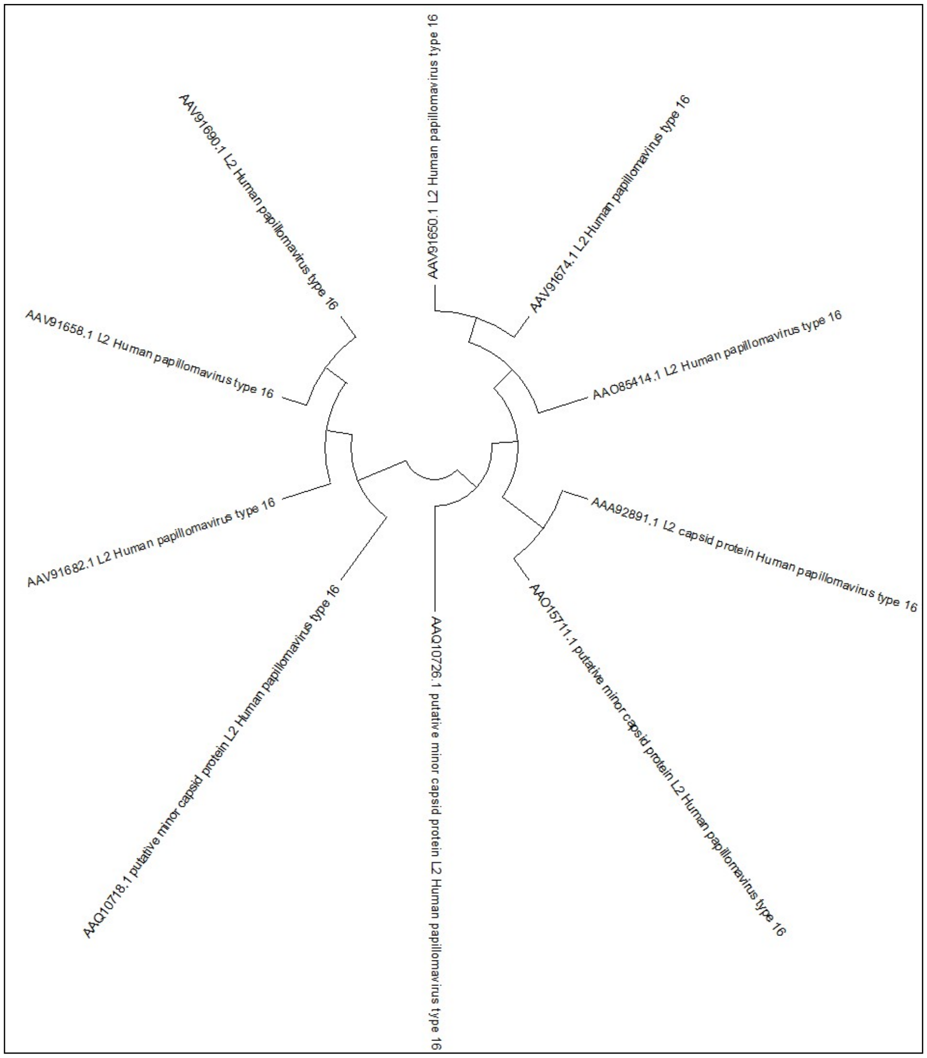

Figure S2. Phylogenetic relationship between the studied protein.

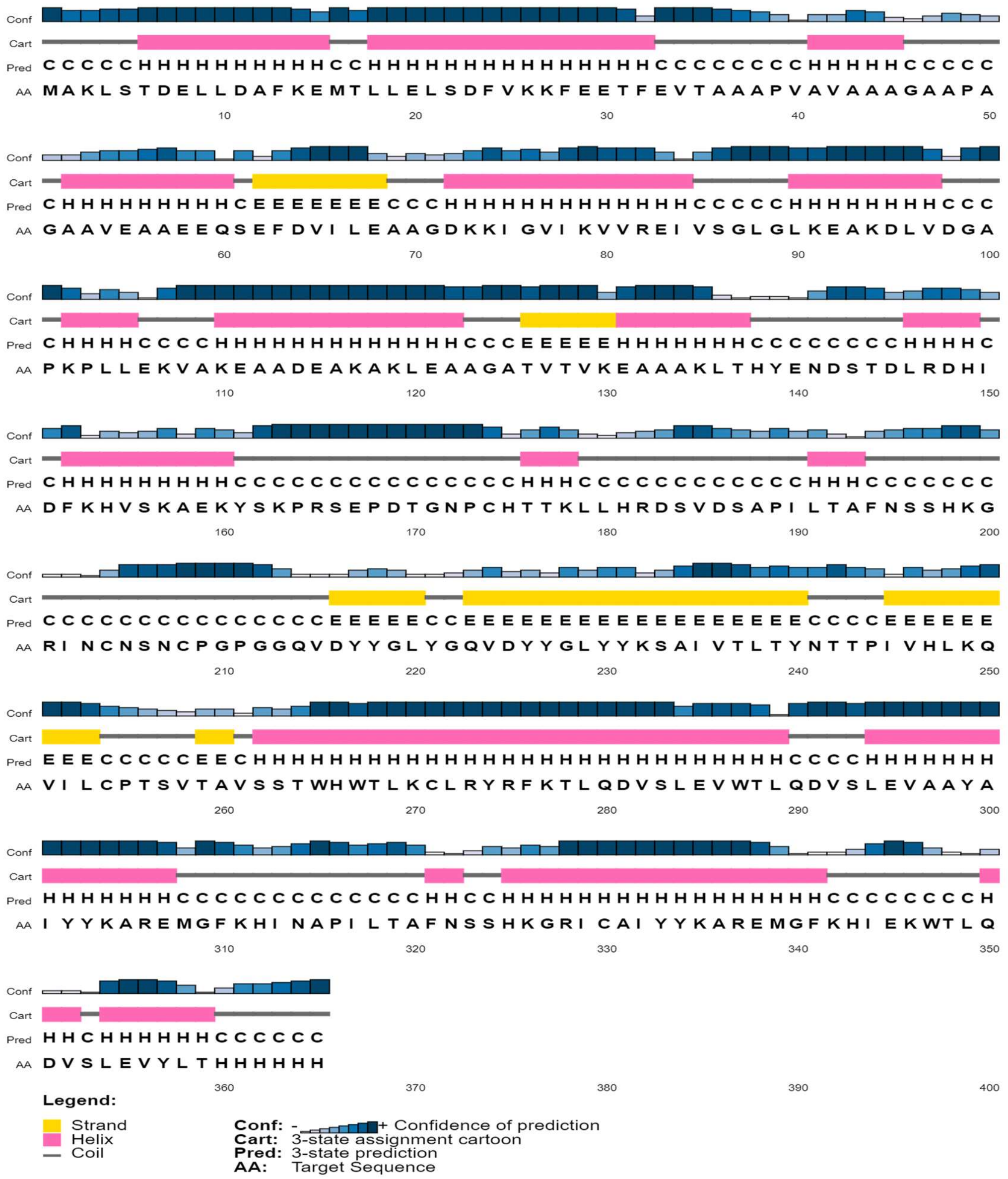

Figure S3. Predicted secondary structure of the chimeric vaccine construct representing strand (yellow), helix (magentas), and coil (gray) colour respectively.

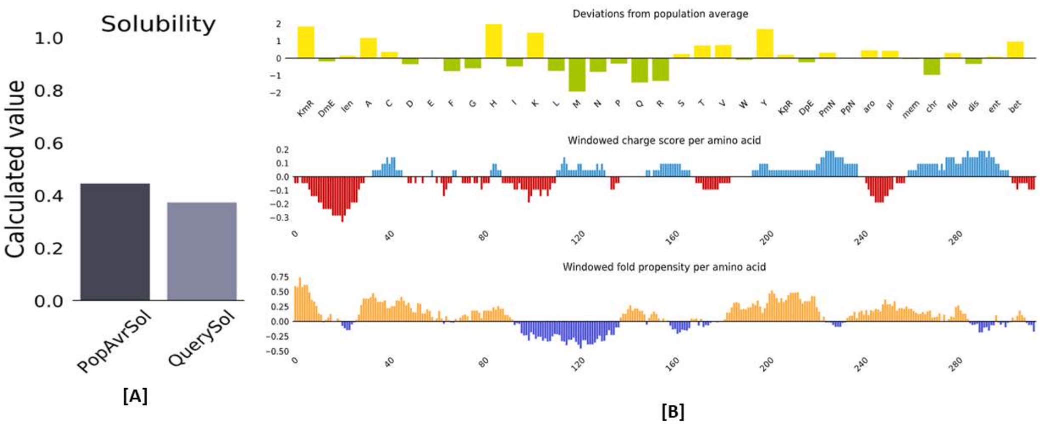

Figure S4. (A) Protein solubility profile of the recombinant vaccine. (B) in addition to its deviation, charge and fold propensity.

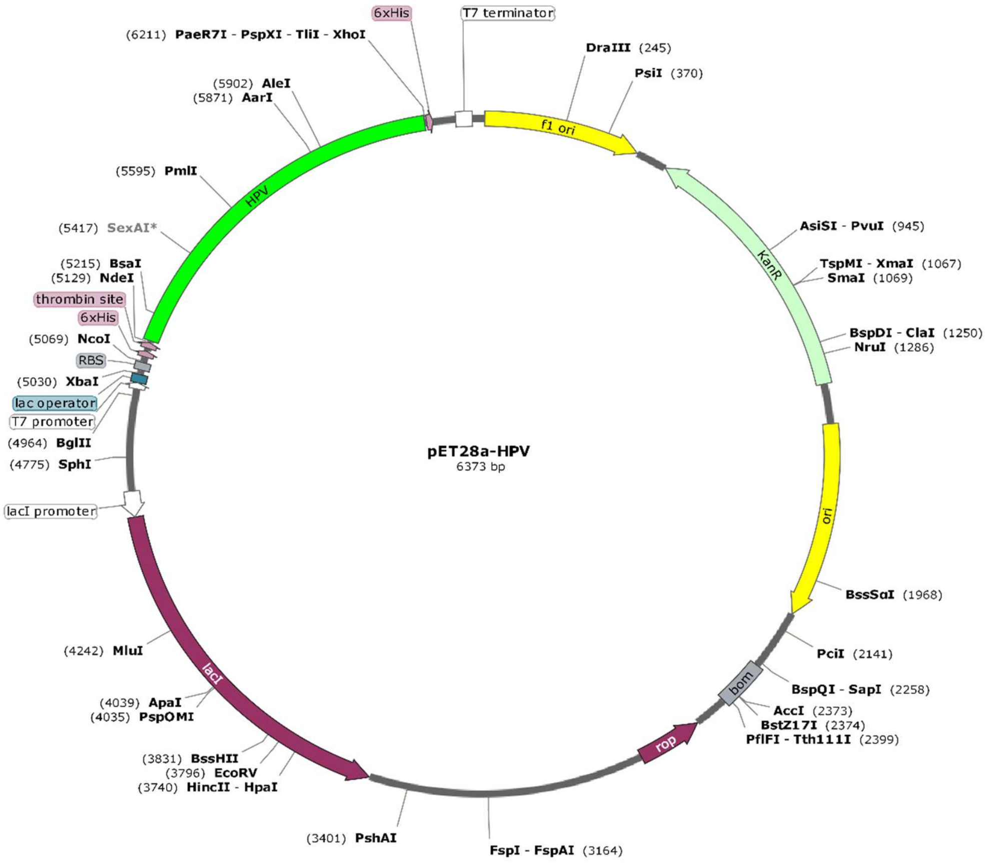

Figure S5. Cloned vaccine in the pET-28a plasmid. The vaccine sequence is shown in green.
